# Hypoxia Augments Outgrowth Endothelial Cell (OEC) Sprouting and Directed Migration in Response to Sphingosine-1-Phosphate (S1P)

**DOI:** 10.1371/journal.pone.0123437

**Published:** 2015-04-15

**Authors:** Priscilla A. Williams, Roberta S. Stilhano, Vivian P. To, Lyndon Tran, Kevin Wong, Eduardo A. Silva

**Affiliations:** 1 Department of Biomedical Engineering, University of California Davis, Davis, California, United States of America; 2 Department of Biophysics, Federal University of São Paulo, São Paulo, Brazil; 3 Department of Neurobiology, Physiology, and Behavior, University of California Davis, Davis, California, United States of America; Medical University Innsbruck, AUSTRIA

## Abstract

Therapeutic angiogenesis provides a promising approach to treat ischemic cardiovascular diseases through the delivery of proangiogenic cells and/or molecules. Outgrowth endothelial cells (OECs) are vascular progenitor cells that are especially suited for therapeutic strategies given their ease of noninvasive isolation from umbilical cord or adult peripheral blood and their potent ability to enhance tissue neovascularization. These cells are recruited to sites of vascular injury or tissue ischemia and directly incorporate within native vascular endothelium to participate in neovessel formation. A better understanding of how OEC activity may be boosted under hypoxia with external stimulation by proangiogenic molecules remains a challenge to improving their therapeutic potential. While vascular endothelial growth factor (VEGF) is widely established as a critical factor for initiating angiogenesis, sphingosine-1-phosphate (S1P), a bioactive lysophospholipid, has recently gained great enthusiasm as a potential mediator in neovascularization strategies. This study tests the hypothesis that hypoxia and the presence of VEGF impact the angiogenic response of OECs to S1P stimulation *in vitro*. We found that hypoxia altered the dynamically regulated S1P receptor 1 (S1PR1) expression on OECs in the presence of S1P (1.0 μM) and/or VEGF (1.3 nM). The combined stimuli of S1P and VEGF together promoted OEC angiogenic activity as assessed by proliferation, wound healing, 3D sprouting, and directed migration under both normoxia and hypoxia. Hypoxia substantially augmented the response to S1P alone, resulting in ~6.5-fold and ~25-fold increases in sprouting and directed migration, respectively. Overall, this report highlights the importance of establishing hypoxic conditions *in vitro *when studying ischemia-related angiogenic strategies employing vascular progenitor cells.

## Introduction

Therapeutic angiogenesis provides a promising approach for clinical treatment of ischemic vascular diseases through regenerative medicine [1–3]. Traditional strategies employ delivery of pharmacological stimuli, recombinant growth factors, or proangiogenic cells to promote new blood vessel formation and restore oxygenation of the hypoxic tissue [1,3–8]. Outgrowth endothelial cells (OECs), also often referred to as endothelial colony forming cells (ECFCs), are vascular progenitor cells that are especially suited as a cell source for treatment of ischemia [7–12]. This purely endothelial subset of endothelial progenitor cells (EPCs) may be noninvasively isolated from adult peripheral or umbilical cord blood for autologous transplantation without immunological barriers. Further, these cells exhibit high telomerase activity, display spontaneous vasculogenesis *in vivo*, are recruited to sites of vascular injury or tissue ischemia after intravenous injection, and directly incorporate within native vascular endothelium to participate in neovessel formation [4,8,12–14]. While hypoxia is known to be a critical aspect involved in microenvironmental regulation of cellular recruitment and angiogenic sprouting [7,15–17], it still remains unclear how OECs respond to external stimulation by soluble cues particularly under hypoxic conditions.

Vascular endothelial growth factor (VEGF) is widely established as a potent factor for initiating new blood vessel formation [1–3,5,18–22]. However, clinical studies involving systemic administration of VEGF have had unsatisfactory results and further studies have highlighted the presence of unstabilized and immature neovessels [1,3–6,19,23]. Therefore, greater knowledge of the interaction between VEGF and other molecules that promote neovessel stabilization in hypoxia is needed. In particular, sphingosine-1-phosphate (S1P) is a naturally occurring, bioactive lysophospholipid that has recently attracted attention in small molecule pharmacological strategies for therapeutic angiogenesis [24–28]. This lipid mediator boasts essential roles in cellular trafficking and recruitment along highly regulated endogenous gradients. S1P has further been touted as a complete angiogenic molecule given its regulatory roles at both early stages of angiogenesis and later stages of neovessel stabilization [18,23,24,29,30]. Bi-directional crosstalk between S1P and VEGF has been proposed [31–33], but the functional nature of these stimuli on OECs in an ischemic microenvironment remains unclear.

Few studies have explored the important relation between hypoxia and S1P and/or VEGF signaling. S1P is carried through circulation largely in forms bound to plasma proteins and is locally released by activated platelets in areas of vascular injury [27]. Overproduced in hypoxia, S1P exerts pleiotropic effects on cellular processes including proliferation, migration, cytoskeletal remodeling, and protection from apoptosis [23,27,32,34]. These effects are largely dependent upon cell type, dose, and S1P receptor (S1PR) expression [23,27,35,36]. Relative S1PR expression is transient and largely dependent upon the presence of extracellular S1P and other growth factors, suggesting the importance of environmental context on the integrated outcome of S1P signaling in a given cell type [25,30,36]. Therefore, in order to effectively deliver S1P as a therapeutic driver of angiogenesis, it is critical to better understand how the cellular microenvironment correlates with local S1P signaling in human cells.

This study tests the hypothesis that reduced oxygen tension and the presence of VEGF impact the angiogenic response of OECs to S1P stimulation *in vitro*. All assays were performed under hypoxia (1% O_2_) and normoxia (ambient) and either with or without the presence of S1P and/or VEGF. The angiogenic response of OECs was tested *in vitro* with different assays that replicate key angiogenic early events including proliferation, wound healing, directed migration towards chemotactic gradients, and 3D sprouting.

## Materials and Methods

### Isolation of outgrowth endothelial cells

Human umbilical cord blood (50–80 cc) was obtained from the UC Davis Umbilical Cord Blood Collection Program (UCBCP) and was used within 12 hours of collection. Written consent was not required for these studies, as all donors are kept anonymous. OECs were isolated from female cord blood following protocols approved by the UC Davis Stem Cell Research Oversight Committee and as previously described [4,10,37]. Blood diluted 1:1 with Hanks balanced salt solution (HBSS; Sigma) was layered over an equivalent volume of Histopaque 1077 (Sigma) and centrifuged for 30 minutes at room temperature. The resultant cord blood mononuclear cell (CBMNC) fraction was collected and treated with red blood cell lysis buffer (eBioscience). The CBMNCs were then cultured on type I collagen-coated tissue culture plates (BD Biosciences) with EGM-2MV medium (Lonza) supplemented with 10% FBS. After 36h, nonadherent cells were removed and the media was changed daily for adherent cells until the first passage. Colonies of OECs appeared between 7 and 21 days of culture. Once a colony grew to the size of a 5X field of view, the cells were detached with 0.05% Trypsin-EDTA (Life Technologies) and plated onto tissue culture treated 6-well plates (Beckton, Dickson and Company (BD)) for continued culture in EGM-2MV. When the cells reached ~80% confluency, they were again detached with 0.05% Trypsin-EDTA and plated onto 25-cm^2^ tissue culture flasks for the first passage (P1) or 75-cm^2^ tissue culture flasks for subsequent passages. OECs were used between P3 and P5 for all experiments.

### Cell culture and cell expansion

EGM-2MV (Lonza) was prepared by supplementing EBM-2 with 5% fetal bovine serum (FBS), ascorbic acid, hydrocortisone, GA-1000 antibiotic, hEGF, VEGF, hFGF-β, and IGF-1 as supplied in the vendor’s kit. N media, defined as EGM-2MV without the addition of the growth factors, was used as the negative control in all experiments. To prepare conditional medias, vascular endothelial growth factor-A (165 isoform) (VEGF) (R&D Systems) and/or sphingosine-1-phosphate (S1P) (Tocris Biosciences) were added to N media at concentrations of 50 ng/mL and/or 1 μM, respectively. S1P was reconstituted as instructed by the manufacturer to create a stock solution at 1 mM in sterile methanol (Sigma) and stored at -20°C [11].

### Hypoxic cell culture

Cells were cultured in a hermetically sealed, modular incubator chamber (Billups-Rothenberg) widely used for hypoxic *in vitro* studies [38,39]. Briefly, the chamber was flushed with a medical grade 1%-O_2_, 5%-CO_2_, 94%-N_2_ gas mixture (Airgas) for three minutes at 30–40 L/min to establish hypoxia according to the manufacturer’s instructions. Humidity was reassured in the chamber by placing a plastic petri dish containing 10 mL of sterile water on the chamber bottom. The media was changed and the chamber was reflushed every 24 hrs.

### Immunocytochemistry (ICC) for human S1PR1

OECs (P5) were seeded in 24-well tissue culture plates (20,000 cells/cm^2^) and allowed to adhere overnight. Hypoxic plates were then transferred to the hypoxia chamber in the incubator for continued culture. After 2 days of culture with daily changes of EGM-2MV medium, the cells were fixed with 4% formaldehyde (Sigma), permeabilized with 0.2% Triton X-100 (Sigma), and blocked with 10% normal goat serum (NGS) (Life Technologies) and 1% bovine serum albumin (BSA; Sigma). Rabbit anti-human EDG-1 polyclonal antibody (Santa Cruz Biotech.) was applied followed with goat anti-rabbit IgG antibody conjugated with Cy3 (Life Technologies). Control wells received 1.5% NGS in DPBS instead of the primary antibody. The cells were imaged at 20X and images were pseudocolor using ImageJ software (NIH).

### Real-time quantitative reverse transcription polymerase chain reaction (qRT-PCR)

OECs (P4) were seeded (5,000 cells/cm^2^) and cultured in EGM-2MV until ~60% confluent. The cells were then treated with conditional media and incubated under either normoxia or hypoxia for 24h. Total RNA was extracted with RNeasy (Qiagen) and treated with DNAse I (Qiagen). cDNA was synthesized using a high capacity cDNA reverse transcription kit (Life Technologies) and qRT-PCR was conducted using QuantiFast SYBR Green RT-PCR Kit (Qiagen) and a Mastercycler RealPlex (Eppendorf). Primers for S1PR1 and β-actin (endogenous reference) were used as previously described [40]. Gene expression relative to the appropriate negative control (N media) in either normoxia or hypoxia was calculated via 2^-ΔΔC^_T_ [11,41]. Each reaction was carried out in duplicate from two or three independent experiments.

### Flow cytometry

OECs (P4) were seeded (5,000 cells/cm^2^) and cultured with EGM-2MV until reaching ~60% confluency. The media was then removed and replaced with the media of interest. For hypoxic studies, the cells were transferred to the hypoxia chamber at this time. EGM-2MV supplemented with 200 μM dimethyloxaloylglycine (DMOG; Sigma) was used as a hypoxia-mimicking control as previously established [42–44]. After 24h, the cells were detached by scraping and mixed with a micropipette to disperse any cellular aggregates. Detached cells were then suspended on a rotating orbital shaker (~1 rev/sec; VWR) at 37°C for 30 minutes under either normoxia or hypoxia to allow for receptor renewal at the cell surface [45]. The cell suspensions were then collected and washed three times (500 x *g* for 5 min at 4°C) with cold, sterile-filtered FACS buffer consisting of 0.5% BSA and 0.1% sodium azide (Sigma) in DPBS. The cells were then put into microcentrifuge tubes, incubated with monoclonal antibodies against human S1PR1 (R&D Systems) for 30 minutes at room temperature, and washed three times with FACS buffer. PE-conjugated anti-mouse secondary antibodies were added for 30 minutes and the cells were again washed three times with FACS buffer. Appropriate controls included unstained cells, cells incubated with the appropriate isotype control (R&D Systems), and cells incubated with just the secondary antibody and no primary antibody. The cells were resuspended in 0.2 mL of FACS buffer and transferred to FACS tubes (BD Falcon) on ice. Flow cytometric analyses were performed using a FACScan cytometer (BD) and data was analyzed using FlowJo (TreeStar Inc., Ashland, OR). A minimum of 10,000 events was analyzed and cells were identified as positive by fluorescence above 95% of isotype control. The percentage of S1PR1+ cells was then calculated and normalized to controls (n = 4).

### Proliferation assays

OECs (P4) were seeded at 10,000 cells/cm^2^ in 6-well tissue culture plates with EGM-2MV and allowed to adhere for 5h. The growth media was then removed and the cells were starved by incubation with serum-free EBM-2 for 16h. After serum deprivation, one of the four conditional medias was added and the cells were either transferred to the hypoxia chamber or retained in normoxia. After 3 days with daily media changes, the cells were detached with 0.05% Trypsin-EDTA. The total number of cells was quantified with a Countess automated cell counter (Life Technologies) and averaged per condition (n = 6). The data was then normalized to the initial cell number based on the seeding density.

### Scratch/wound healing assays

A modified wound-healing assay was conducted to assay cellular motility in response to the various stimuli [46,47]. OECs (P4) were seeded (10,000 cells/cm^2^) and cultured in EGM-2MV medium until reaching ~80% confluency. The cells were then starved by incubation with serum-free EBM-2 medium for 16 hrs. A scratch was made manually down the center of the well with a p200 micropipette tip in one swift motion as previously described [46]. One of the four conditional medias was applied and pictures (10X) were taken at two different locations along the scratch wound immediately after scratching [46]. The cells were incubated either under ambient oxygen tension or within the hypoxia chamber for 12 hours, washed twice with DPBS++, and then fixed overnight in 4% formaldehyde (Sigma) at 4°C. Pictures were then taken at the same locations along the scratched wound that were imaged immediately after scratching. Wound closure was quantified as the difference in scratch width between the initial and final images at 10X using two pictures per well. The data was then normalized to the average value for the respective negative control in either normoxia or hypoxia (n = 5).

### Sprouting assays

Cytodex 3 microcarrier (MC) beads (GE Healthcare Life Sciences) were hydrated overnight in DPBS at room temperature with gentle agitation and sterilized with autoclaving. After cooling, the sterile MC beads were mixed with 1.5 mL of OECs (~5 x 10^6^ P4 cells) in EGM-2MV medium, transferred to a FACS tube, and incubated at 37°C with gentle agitation made by inverting the tube 3–5 times every 20 minutes for 4h. The cell-laden MC beads were then cultured in T25 flasks until nearly 100% confluent on a rotating orbital shaker (~1 rev/sec) at 37°C with daily media changes. For analysis of 3D sprout formation, the cell-laden MC beads were incorporated within fibrin gels as previously described with minor modifications [4,48,49]. Briefly, beads suspended in N media were combined with fibrinogen (Sigma) solution (4 mg/mL in 0.9% NaCl) supplemented with aprotinin (Sigma; ~60 μg/mL) and distributed in 24-well plates. A second solution containing thrombin (Sigma; 2.1 U/mL in DPBS) was then added at a 4:5 ratio and the plates were incubated at 37°C for 30 minutes. Gels were then topped with one of the four conditional medias and incubated under either normoxia or hypoxia for 1 day. The gels were subsequently washed with DPBS and fixed overnight at 4°C in 4% formaldehyde. For fluorescent imaging, the cells were stained with Hoescht 33342 (nuclei; Life Technologies) and Rhodamine R18 (cell membrane; Life Technologies). The total number of beads (n_T_), empty beads (n_E_), and sprouts (n_S_) was manually quantified per well. A sprout was defined as more than one EC migrating outwards linearly while remaining anchored to the bead. The average number of sprouts per bead in each gel, n_ave_ = n_S_/(n_T_-n_E_), was then calculated and normalized to the average value for the respective negative control wells in either normoxia or hypoxia (n = 4). Representative images of sprout formation were taken at 10X.

### Alginate hydrogel formulation

Ultra-pure (UP) alginate polymer was purchased from ProNova Biomedical. MVG alginate containing a higher G-block content (~ 60% as specified by the manufacturer) was used as the high molecular weight (HMW; ~250 kDa) component to prepare gels. Low molecular weight (LMW) alginate (~50 kDa) was obtained by gamma (γ)-irradiating the HMW UP MVG alginate as previously described [19,48]. Both low and high MW alginate polymers were oxidized with sodium periodate (Sigma) to an extension of 1% of the sugar residues in the polymer [19,48]. The oxidized alginate solutions were dialysed, sterile filtered, lyophilized, and subsequently stored at -20°C. To prepare gels, modified alginates were reconstituted to 2% w/v in EBM (Lonza). For S1P- and/or VEGF-loaded gels, 15 μM S1P and/or ~1.3 μg/mL VEGF was loaded in alginate solutions prior to addition of calcium cross-linking. A 75:25 LMW:HMW ratio of alginate solutions was ionically crosslinked with a calcium sulfate slurry (Sigma) in a ratio of 25:1 (40 μl of CaSO_4_ per 1 ml of 2% w/v alginate solution). Alginate hydrogel was dispensed onto a sterile glass plate set with 1-mm spacers, sandwiched with another sterile glass plate, and incubated for 30 minutes (at room temperature) to ensure full gelation.

### Release kinetics from alginate hydrogels

Alginate hydrogels loaded with S1P were immersed in a physiological eluent buffer consisting of EBM supplemented with 5% FBS (1 mL buffer per mL of alginate) and incubated at 37°C. Additionally, alginate hydrogels loaded with VEGF_165_ were immersed in a physiological eluent buffer consisting of DPBS++ (1 mL buffer per mL of alginate). Samples of buffer were collected at various time points and the tubes were replenished with fresh buffer. The amounts of S1P and VEGF released were quantified by ELISA following the manufacturers’ instructions (Echelon for S1P and R&D System for VEGF_165_). Samples taken from blank alginate hydrogels immersed in buffer were used to subtract background readings of S1P from the FBS content at each time point (n = 4).

### Directed migration assays

A modified Transwell chemotaxis assay was used to assay 3D matrix invasion and directed migration of OECs (P4) *in vitro*. Transwell inserts (5 μm pores; Corning Inc.) were soaked in EBM-2 overnight to prime the filter membranes. Bimodal 75:25 (LMW:HMW) alginate hydrogels loaded with S1P and/or VEGF were prepared. Blank alginate hydrogels served as the negative control. Alginate disks were punched out using a 10-mm biopsy punch (Acuderm Inc.), placed at the bottom of each well of a 24-well plate and then covered with 0.5 mL of fibrin gel solution. After 40–50 minutes of incubation at 37°C, 0.5 mL of N media was added on top of the fibrin gel. A pre-incubated Transwell insert was then placed within the well, making slight contact with the fibrin gel, and 30,000 cells were seeded into the insert in 100 μL of N media. The plates were incubated under either normoxia or hypoxia for 48h and the inserts were then removed using sterile tweezers. The gels were rinsed twice with DPBS++ and then fixed with 2% formaldehyde (4% formaldehyde diluted 1:1 with DPBS++) overnight at 4°C. The total number of cells present in six representative 20X images of the gels was reported and averaged for each condition (n = 3).

### Statistical analysis

Results are shown as the mean values with standard deviations. Comparisons were assessed by Student’s unpaired t-tests. Differences between conditions were considered significant if P<0.05. All analyses were performed using GraphPad Prism software (GraphPad Software Inc.).

## Results

### OEC expression of S1PR1 is altered by microenvironment

**Fig 1:**
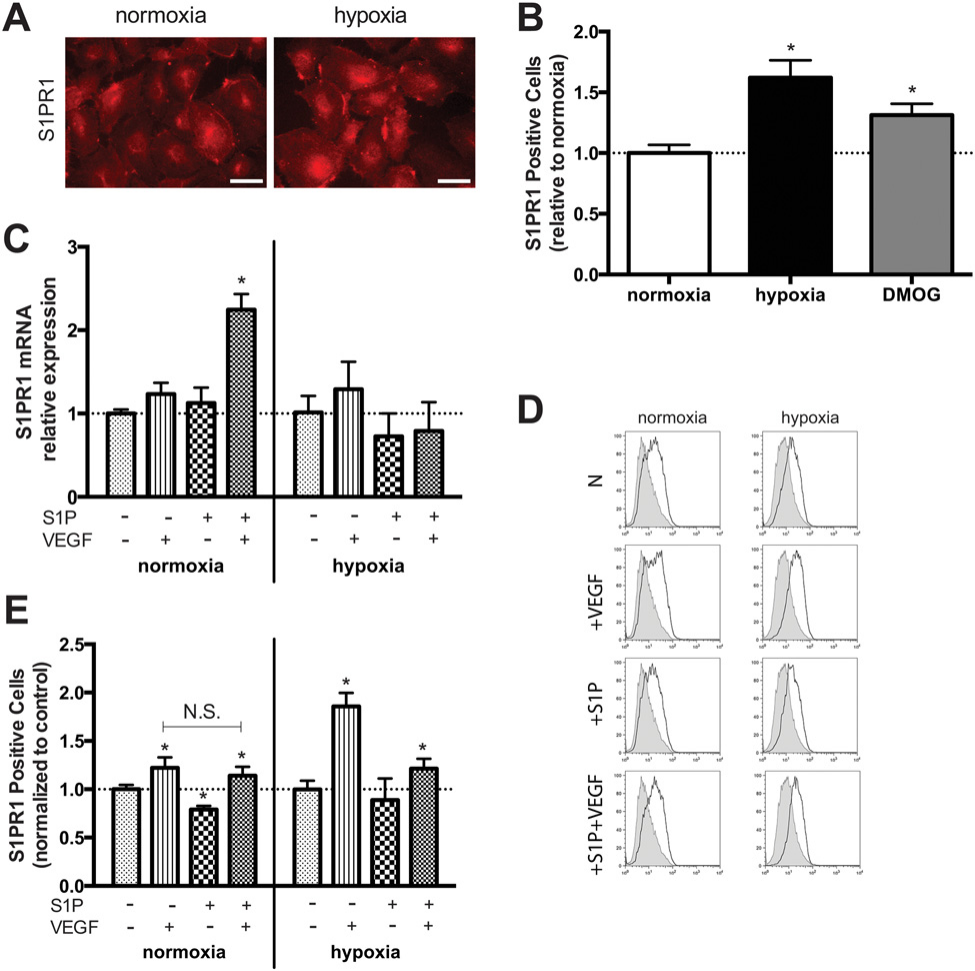
S1PR1 expression by OECs is dynamically altered by microenvironment. OEC expression of S1PR1 under normoxia and hypoxia was qualitatively confirmed by immunocytochemistry (A). The percentage of cells expressing S1PR1 on the cell surface, as confirmed with flow cytometry, was enhanced under hypoxia as compared to the normoxic control (B). Combined stimuli of S1P and VEGF resulted in greater S1PR1 mRNA production after 24h in normoxia (C). Combined stimuli also resulted in a greater proportion of S1PR1+ OECs under both normoxia and hypoxia (D and E). Scale bars represent 40 μm. Data are mean ± SD (n = 4) and normalized to the average untreated control values for either normoxia or hypoxia (C, E; indicated by dashed line) accordingly. An asterisk indicates statistically significant differences (P<0.05) between conditions in normoxia or hypoxia respectively and N.S. displays no statistically significant difference between conditions.

To evaluate the presence of S1PR1 on OECs, cells were stimulated with S1P and/or VEGF both in normoxic and hypoxic conditions. The S1PR1 expression was analyzed with immunocytochemistry, real-time RT-PCR, and flow cytometry. ICC qualitatively confirmed that OECs express S1PR1 under both normoxia and hypoxia (Fig 1A). Additionally, flow cytometry revealed that the percentage of OECs that express S1PR1 on the cellular surface is increased ~1.6 fold under hypoxia as compared to normoxia (Fig 1B). This increased expression under hypoxia was also observed in OECs cultured with EGM-2MV containing DMOG (hypoxic control). While neither S1P nor VEGF augmented S1PR1 mRNA production, the combination of both stimuli resulted in greater levels of expression under normoxia (Fig 1C). Interestingly, this combinatory effect was not observed under hypoxia. The percentage of OECs expressing S1PR1 was significantly increased in both normoxia and hypoxia in the presence of VEGF with or without S1P (Fig 1D and 1E). The percentage of S1PR1+ cells was reduced in the presence of S1P alone under normoxia, but this effect was abrogated under hypoxia. Further, percentages were significantly increased by VEGF stimulation under hypoxia as compared to that under normoxia.

### The combination of S1P and VEGF results in greater proliferation of OECs

**Fig 2:**
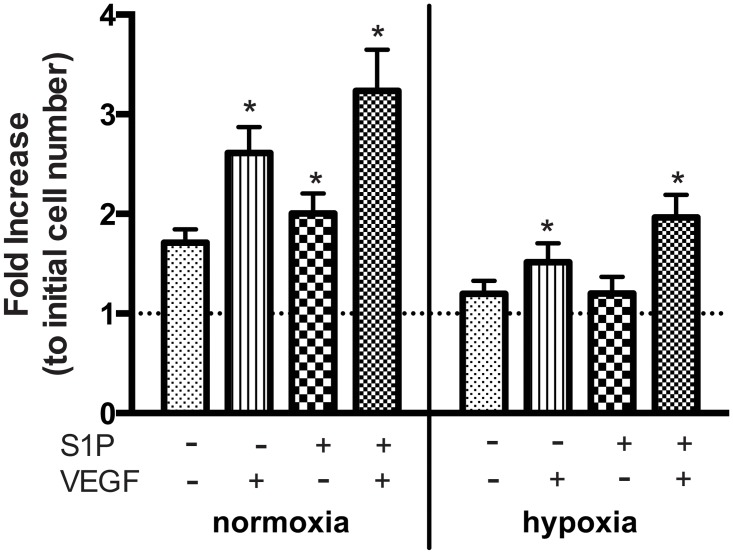
The combination of S1P and VEGF induced greater OEC proliferation than either individual stimulus under both normoxia and hypoxia. Data are mean ± SD (n = 6) and normalized to the initial cell seeding number (indicated by dashed line). An asterisk indicates statistically significant differences (P<0.05) between conditions in normoxia or hypoxia respectively.

The proliferative effect of 1 μM S1P and/or 50 ng/mL VEGF on OECs under normoxia and hypoxia was evaluated after 3 days of stimulation. VEGF resulted in approximately 1.5-fold and 1.3-fold increases in OEC proliferation under normoxia and hypoxia, respectively (Fig 2). While S1P resulted in about 1.2-fold more proliferation under normoxia, this effect was abolished under hypoxia. Interestingly, the combined stimuli of both S1P and VEGF led to the greatest degree of proliferation under both oxygen tensions.

### Wound healing is accelerated in response to S1P and VEGF

**Fig 3:**
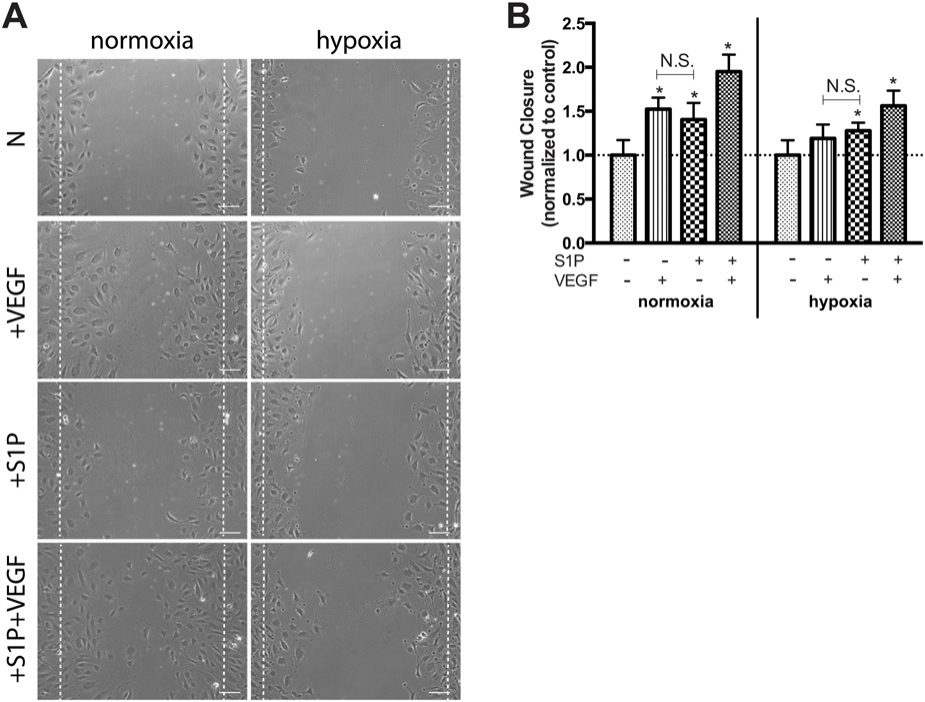
Combination therapy enhanced S1P-induced motility under both normoxia and hypoxia. OEC motility was assessed in response to 1.0 μM S1P and/or 50 ng/mL VEGF for 12h under normoxia and hypoxia (A). S1P and VEGF combined resulted in accelerated wound closure compared to either stimuli alone under both normoxia and hypoxia (B). Data are mean ± SD (n = 5) and normalized to the average untreated control values for either normoxia or hypoxia (indicated by the dashed line) accordingly. An asterisk indicates statistically significant differences (P<0.05) between conditions in normoxia or hypoxia respectively and N.S. displays no statistically significant difference between conditions.

The ability of S1P and/or VEGF to accelerate OEC wound closure was investigated under both normoxia and hypoxia in a scratch/wound assay (Fig 3A). Individual stimuli of either S1P or VEGF resulted in similar degrees of accelerated wound closure versus control in normoxia (Fig 3B). VEGF also induced OEC motility under hypoxia to a similar degree as S1P, although not statistically significant from control. S1P and VEGF together resulted in maximum rates of wound closure under both normoxia and hypoxia, with near complete closure in normoxia after only 12 hours.

### Hypoxia augments 3D sprouting formation in response to S1P

**Fig 4:**
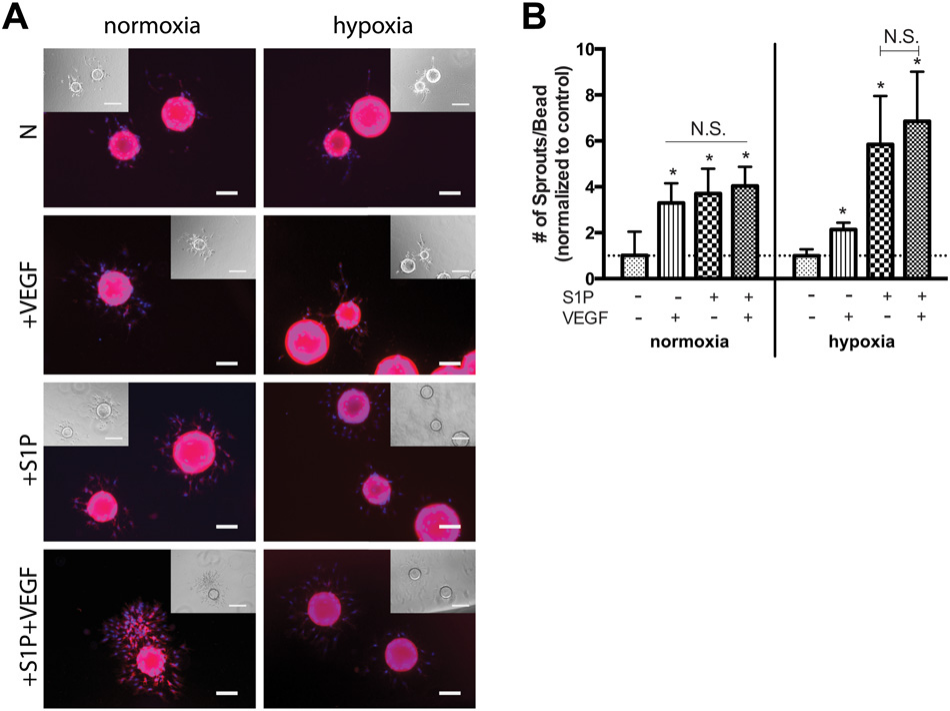
Hypoxia augmented the angiogenic response of OECs to S1P stimulation in terms of 3D sprouting formation. S1P and/or VEGF induced OEC sprouting in a fibrin gel after 1 day of culture in normoxia and hypoxia (A). S1P alone or in combination with VEGF led to significantly more sprouts per bead than VEGF alone under hypoxia (B). Scale bars represent 100 μm and 200 μm (DIC inlay). Data are mean ± SD (n = 4) and normalized to the average untreated control values for either normoxia or hypoxia (indicated by the dashed line) accordingly. An asterisk indicates statistically significant differences (P<0.05) between conditions in normoxia or hypoxia respectively and N.S. displays no statistically significant difference between conditions.

The ability of OECs to form sprouts was used to evaluate the functional angiogenic response to S1P and/or VEGF under both normoxia and hypoxia (Fig 4A). Interestingly, the presence of S1P or VEGF either alone or in combination resulted in equal degrees of enhanced sprouting formation (about 3–4 fold) under normoxia (Fig 4B). Strikingly, while VEGF resulted in only ~2-fold more sprouts/bead than control under hypoxia, co-stimulation with S1P drastically enhanced sprouting by ~7-fold versus control. Additionally, stimulation by S1P alone resulted in significantly more sprouts/bead than by VEGF alone under hypoxia; this enhanced effect of S1P was of equal magnitude to that induced by S1P and VEGF combined.

### Directed migration is enhanced by S1P under hypoxia

**Fig 5:**
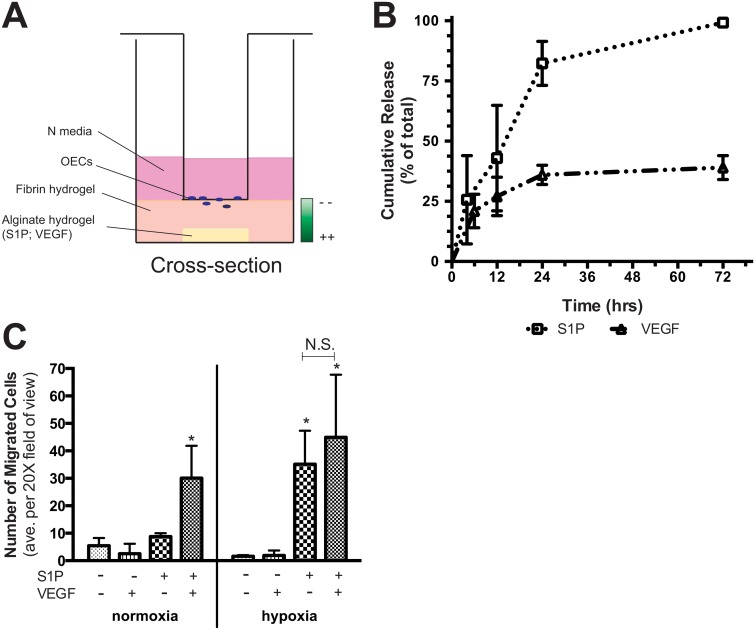
Hypoxic culture led to enhanced directed migration towards a gradient of S1P *in vitro*. A modified transwell assay was used to quantify directed migration/3D matrix invasion of OECs towards S1P in a fibrin gel (A). Gradients of S1P and VEGF were established by sustained delivery from an alginate hydrogel (B). While combined release of S1P and VEGF resulted in greater migration after 48h in both normoxia and hypoxia, S1P alone was equally sufficient under hypoxia (C). Data are mean ± SD (n = 4). An asterisk indicates statistically significant differences (P<0.05) between conditions in normoxia or hypoxia respectively and N.S. displays no statistically significant difference between conditions.

A modified Transwell chemotaxis assay (Fig 5A diagram) was designed and tested to evaluate directed migration and 3D matrix invasion of OECs towards sustained gradient(s) of S1P and/or VEGF. Quantification of S1P and VEGF release from alginate hydrogels into an aqueous eluent buffer revealed that sustained release was achieved with roughly 80% and 40% total release, respectively, during the first 24 hours (Fig 5B). After 48h under either normoxia or hypoxia, the combination of S1P and VEGF resulted in greater numbers of OECs infiltrated into a fibrin gel that lay below the Transwell insert (Fig 5C). Interestingly, stimulation by S1P alone resulted in as much migration as S1P and VEGF combined specifically under hypoxia, whereas there was no effect under normoxia. In contrast, VEGF alone did not induce directed migration versus control under either normoxia or hypoxia.

## Discussion

The results of this study revealed that the capacity for S1P to induce angiogenesis by OECs is largely dependent upon oxygen tension and VEGF presence. To our knowledge, the potential of S1P to stimulate vascular progenitor cells in hypoxia has never been tested before *in vitro*. Given that reduced oxygen tension is a clinically relevant condition of tissue ischemia, we demonstrated the angiogenic response of OECs to S1P (1 μM) particularly under hypoxia versus normoxia. We established that the additional co-stimulation with VEGF (50 ng/mL) resulted in a pronounced increase of OEC response, including proliferation, wound healing, sprouting, and directed migration, regardless of oxygen tension. Remarkably, hypoxic culture enhanced the response of OECs to S1P alone to a degree that was markedly as effective as S1P and VEGF together in terms of directed migration and sprouting. Altogether, we highlight the impact of environmental context on S1P-regulated angiogenesis by a prominent vascular progenitor cell involved in postnatal vascularization.

We showed that OECs express S1P receptor 1 (S1PR1), a prominent S1PR involved in the stimulatory angiogenic effects that S1P elicits in endothelial cells [11,50,51]. Previous work has also shown that both human cord blood derived ECFCs [11] and peripheral blood derived EPCs [51] express S1PR1 under various culture conditions under normoxia, but very little is known about expression of S1PR1 under hypoxia. Interestingly, we observed that hypoxic conditions in growth media increased the overall percentage of OECs expressing S1PR1 on the cell surface.

OEC stimulation by exogenous VEGF with or without S1P also resulted in an enhanced portion of OECs expressing S1PR1. This VEGF-induced effect was further increased ~1.6-fold under hypoxia as compared to normoxia although the additional presence of S1P hindered this response. This dynamic expression observed on OECs could be attributed to the fact that S1PR expression is transient and largely dependent upon the presence of extracellular S1P and other growth factors [25,30,36]. Accordingly, VEGF activation of sphingosine kinase-1 (SK1) promotes production of S1P [32] and VEGF stimulation of bovine aortic ECs has been shown to enhance S1PR1 expression [34]. Further, previous work has also shown that S1PR1 mRNA production is upregulated *in vivo* in a hypoxia-induced mouse model of pathologic retinal angiogenesis; this hypoxia-induced increase in S1PR1 correlated with an increase in VEGF mRNA production as well [52]. Our studies also revealed a lower percentage of S1PR1+ OECs in the presence of S1P stimulation alone, which accordingly may be due to receptor internalization upon activation by S1P binding [30]. The discrepancies between our RT-PCR and flow cytometry based findings may be explained by the time delay between mRNA expression and protein production/translocation to the plasma membrane [53]. However, our overall data reveals that hypoxia alters S1PR1 expression, dynamically regulated in human OECs by the presence S1P and/or VEGF, and promotes the importance of considering microenvironmental cues when studying S1P for angiogenic applications.

The combination of S1P and VEGF maximally promoted early angiogenic activity assessed via proliferation and motility (wound healing) under both normoxia and hypoxia. These two processes are crucial for enabling vessel wall remodeling and initiating angiogenesis [18,54]. We found that 1 μM S1P was sufficient to promote OEC proliferation under normoxia in accordance with previous studies using human microvascular endothelial cells (HMVECs) [55], umbilical vein endothelial cells (HUVECs) [56], and endothelial colony forming cells (ECFCs) [11]. However, we further demonstrated that this proliferative response to S1P was not maintained under hypoxia unless VEGF (50 ng/mL) was additionally present. In contrast, S1P stimulation accelerated OEC wound healing under both normoxia and hypoxia. Interestingly, while OECs were more motile in response to VEGF under normoxia, this effect was not observed under hypoxia. These overall findings, which highlight the compounded effect of dual stimulation by S1P and VEGF under both normoxia and hypoxia in the early angiogenic activity of OECs, have not been shown before to our knowledge.

Dual stimulation was uniquely not required in order to induce functional angiogenic responses (sprouting, directed migration) of OECs to S1P stimulation although the combination of stimuli did still have a positive effect. This finding is in contrast with a recent study, where it was shown that combined stimulation with S1P and VEGF resulted in lower sprouting than VEGF alone in mouse pancreatic islet-derived microvascular endothelial (MS-1) cell-covered beads in fibrin gels after 6 days [31]. However, the study was conducted with an additional feeder layer of human fibroblasts, which may have confounded the response to the indicated stimuli. Interestingly, we revealed that VEGF or S1P resulted in similar levels (almost 4-fold increase) of OEC sprouting in fibrin gels under normoxia and these responses were decreased or increased, respectively, under hypoxia. Previous reports have separately shown that both VEGF [8,19,48,49,57] and S1P [11,57] individually promote sprouting of capillary-like structures *in vitro*, but this is the first time to our knowledge that the responses were shown to be oppositely altered by hypoxic conditions. Interestingly, S1P remarkably enhanced both sprout formation (~6.5-fold) and directed migration (~25-fold) whether given alone or in conjunction with VEGF under hypoxia. However, both S1P and VEGF were conversely required to induce directed migration under normoxia. In contrast, previous reports have shown that 1 μM S1P [11] and 50 ng/mL VEGF [8] each individually induce OEC chemotaxis after 4 hours under normoxia. This discrepancy may be attributed to the fact that, in the biomaterial based method developed within this manuscript, factors are presented as a temporally sustained gradient that is not established in traditional chemotaxis assays. Indeed, it has been recognized that the nature of the surrounding matrix greatly alters the migratory response [58,59], as observed in the differing magnitude of the degree of motility versus directed migration that was induced by S1P stimulation. Herein, we demonstrated overall that hypoxia positively augmented the functional angiogenic responses of OECs to S1P stimulation in contrast to early angiogenic processes of proliferation and motility.

These studies involving the presence of VEGF and low oxygen tension confirmed the hypothesized dependency of S1P-regulated angiogenesis by OECs on environmental context. These results provide urgency towards the need for establishing hypoxic conditions *in vitro* that mimic the diseased microenvironment *in vivo* in order to accurately glean information about the therapeutic potential of S1P as an angiogenic mediator in future studies. Altogether, our results suggest the potential future for delivering S1P in a localized, spatiotemporally controlled manner within ischemic tissue for local recruitment and enhanced stimulation of circulating vascular progenitor cells for therapeutic angiogenesis.
